# In vitro and in vivo effects of the PPAR-alpha agonists fenofibrate and retinoic acid in endometrial cancer

**DOI:** 10.1186/1476-4598-5-13

**Published:** 2006-03-28

**Authors:** Samir A Saidi, Cathrine M Holland, D Stephen Charnock-Jones, Stephen K Smith

**Affiliations:** 1 University Department of Obstetrics & Gynaecology, The Rosie Hospital, Robinson Way, Cambridge, CB2 2SW, UK

## Abstract

Fenofibrate, an agonist of PPAR-alpha, in doses above 25 μM, inhibits proliferation and induces apoptosis in Ishikawa endometrial cancer cells. We show that these effects are potentiated by retinoic acid, an agonist of the retinoid-X-receptor. DNA content analysis shows that G_1_/S phase progression through the cell cycle is inhibited. Independent Component Analysis of gene microarray experiments demonstrated downregulation of Cyclin D1 (CCND1) and associated changes in cell cycle gene expression. Expression of PPAR-alpha mRNA was reduced by >75% using RNA-interference but this resulted in only minor changes in biological effects. A nude mouse model of endometrial carcinoma was used to investigate the effect of fenofibrate *in vivo *but failed to show consistent inhibition of tumour growth.

The combination of fenofibrate and retinoic acid is a potent inhibitor of Ishikawa endometrial cancer cell growth *in vitro*.

## Introduction

Endometrial cancer is the most prevalent gynaecological cancer in the UK, and represents the third commonest cancer affecting women in the Western World [1]. By contrast, the incidence in the non-Western world is approximately ten-fold lower [2]. The excellent prognosis of early stage endometrial cancers belies the impact of the disease on mortality, being of similar magnitude to that of cervical cancer [2]. Indeed, the long-term survival of advanced stage endometrial cancer, at approximately 10%, is similar to that of ovarian cancer.

Established risk factors for sporadic endometrial cancer mainly involve hormonal factors, with the unopposed estrogen hypothesis believed to be the central pathogenetic mechanism [3,4]. Although this theory is strongly supported, it does not satisfactorily account for all the risk factors associated with endometrial cancer risk. Obesity is a significant independent risk factor, with relative risks in the 2–10 range [5,6]. The mechanism for this has not yet been elucidated but postulates include the collateral involvement of estrogen and insulin-like growth factor (IGF) receptor pathways [5,7]. Improving understanding of the carcinogenesis of endometrial cancer is essential in the development of targeted therapy.

The potential of gene array methods and systems biology has been exploited in recent years for the investigation of a number of tumour types [8,9]. The aim of the new biology is to provide a global overview of carcinoma at the molecular level, whilst focusing on biologically relevant data. Although oncology has received a great deal of attention from computational biology, a limited number of gene array studies have been applied solely to endometrial cancer [10-12].

Using gene array methods within a computational biology environment, we have previously demonstrated that lipid metabolism is likely to play an important role in endometrial carcinogenesis [12,13]. Consequentially, we identified fenofibrate, a ligand of the peroxisome proliferator-activated receptor alpha (PPARα), as a potential therapeutic agent in endometrial cancer [12]. PPARs comprise a group of transcription factors belonging to the nuclear hormone receptor subfamily and consist of subtypes α, γ and β/δ [14]. Their main actions regulate the metabolism of fatty acids and are therefore closely involved with prostanoid pathways [14]. Furthermore, receptor-mediated transcription is dependent upon heterodimerisation with the retinoid-X receptors (RXRs). Following activation by their ligands (eg fenofibrate and fatty acids in the case of PPARα) and heterodimerization with RXR, PPARs bind to the peroxisome-proliferator response element (PPRE) in the promoter of their target genes and activate their transcription [14]. PPREs are most commonly found in genes that are involved in lipid metabolism and energy homeostasis, including lipid storage or catabolism (β-oxidation and ω-oxidation), fatty-acid transport, uptake and intracellular binding. In recent years there has been interest and some success in the use of retinoids, synthetic ligands of the RXR, in the treatment of hormonally derived cancers such as those of the breast and endometrium [15,16].

Our previous work demonstrated upregulation of PPARα transcript in association with downregulation of its heterodimerisation partner RXRβ [12,13] in endometrial cancer. We also showed that the PPARα agonist fenofibrate, in doses above 25 μM, inhibits Ishikawa and ECC-1 endometrial cancer cell growth in vitro, in association with increased apoptosis and PPARα receptor activation [12]. In this study, attention was focussed on the Ishikawa cell line in view of its endometrioid-like characteristics, estrogen receptor positivity [17] and suitability for xenografting [18].

Having identified PPARα as a potential therapeutic target in endometrial cancer, the aim of this study was to further investigate the biological effects of fenofibrate, from a molecular to a cellular level and finally to an animal model. We further aimed to investigate whether targeting the PPARα receptor using retinoid-X-receptor ligands would increase the growth-inhibitory effects of this agent. Finally, a systems biology approach was used to help understand the mode of action of fenofibrate by identifying the global transcription changes induced in the treatment of endometrial cancer *in vitro*.

## Materials and methods

### In vitro studies

#### Cell culture & proliferation assays

Ishikawa cells were obtained from the European Collection of Cell Cultures (Cat. No. 99040201) [19] and were grown in DMEM/F-12 Ham medium (Cat. No. D6421, Sigma-Aldrich, UK) supplemented with L-glutamine and 10% fetal calf serum in 96-well plates (proliferation assays), 6-well plates (FACS analysis, luciferase reporter assays) or cell culture flasks (RNA extraction, tumour explant preparation). Cells were cultured at 37°C and 5%CO_2 _with varying doses of fenofibrate (Cat. No. F6020), 9-cis retinoic acid (9-cRA) (Cat. No. R4643) or all-trans retinoic acid (ATRA) (Cat. No. R6265, all Sigma-Aldrich, UK) dissolved in DMSO (Sigma-Aldrich). Control cells were treated with DMSO and the concentration of DMSO was kept the same throughout each experiment, to a maximum of 1% v/v. A minimum of five replicates per dose was performed for automated assays (BrdU and MTS) and all other cell culture experiments were performed independently in duplicate or triplicate. Cell proliferation was measured by the uptake of 5-bromo-2'-deoxyuridine (BrdU) using the BrdU Labelling and Detection Kit (Cat. No. 1444611, Roche, UK) according to the manufacturer's instructions. Relative cell abundance was measured either by cell counting, or using an MTS assay (CellTiter 96 Aqueous Cell Proliferation Assay, Cat. No. TB169, Promega, UK). Absorbance values for each well were measured using a microtiter plate reader (Anthos Labtech Instruments, Salzburg, Austria)

#### FACS analysis

All FACS analysis was performed on a FACSCalibur analyser (Becton Dickinson, USA). Computational analysis was performed using FCSPress software for Macintosh [20]. Gating parameters were based on untreated cells in each experiment, to exclude subcellular fragments and conglomerate cells.

#### Apoptosis analysis

Cells in 6-well plates at approximately 80% confluence were washed in PBS, and the medium replaced with medium containing DMSO (control) or drug in DMSO solution to a maximum of 1% v/v DMSO. After 3 hours' exposure, the cells were harvested by trypsinisation, spun, and resuspended in 500 μl buffer (containing per ml of serum-free medium: 4 μl Propidium Iodide (Cat. No. P3566, Invitrogen, USA), 3.5 μl Annexin-V FITC (Cat. No. A13199, Invitrogen, USA), 10 μl 0.1 M Calcium chloride). Cells were transported on ice and FACS analysis was performed within one hour.

#### DNA content analysis

Cells were similarly treated with drug solution for 12 hours, and harvested as above. Approximately 10^6 ^cells were fixed in 0.3 ml of PBS and 0.7 ml of ice cold 70% EtOH at 4°C for 1 hr. After fixation, the cells were resuspended in 0.25 ml of PBS containing 12.5 μl of PI and 6.25 μl RNase A (20 mg/ml, SIGMA, UK). Cells were incubated at 37°C for 15 minutes prior to FACS analysis.

#### Cell counting

Cell counting was independently verified using FACS analysis. Samples harvested by trypsinisation were fixed in ethanol as above. 20 μl of a 1:1000 aqueous solution of Fluoresbrite plain YG 2 μm beads (Cat. No. 18338, Polysciences Inc, Germany) was added to 1 ml of cells resuspended in PBS containing 10 μl Propidium Iodide solution, giving a final bead concentration of 5.68 × 10^5^/ml. Cell concentration was then calculated as: (Cell count) × (Bead Count)^-1 ^× (5.68 × 10^5^)

#### RNA interference

RNAi was performed through a lipid-based transfection method, with either PPARα-RNAi (Cat. No. M-003434-00) or Control-RNAi (Cat. No. D-001206-13), using SmartPool reagents (Dharmacon, Lafayette CO, USA) according to the manufacturers' instructions. Cells were harvested at confluence from a 75 cm^2 ^culture flask, and plated at approximately 2 × 10^6 ^cells per plate into 6 well plates with 2 ml/well of medium as described. For transfection, 5 μl/well of 20 μM RNAi (control or PPAR), 4 μl/well of lipofectamine 2000 (Cat. No. 18324-111, Invitrogen, UK) and 100 μl/well of serum-free medium were incubated at room temperature. 100 μl/well of transfection mixture was then added to each well for overnight transfection, giving a transfection rate ≥30%, as measured by beta-galactosidase staining. The medium was replaced with fresh medium and cells grown to confluence. Cells were then harvested and pooled in each group (control RNAi/PPAR RNAi) before replating to 6-well plates. Cells later harvested for RT-PCR analysis were plated from the same batches. For cell counting, cells were allowed to grow in drug-containing media for 48 hours, then harvested. For FACS analysis (apoptosis assays), drug-based media was added to cells for 2 hours before harvesting.

### Microarray studies

#### RNA preparation

Cells in the logarithmic growth phase were plated into 75 cm^2 ^culture flasks containing culture medium as described, containing DMSO or fenofibrate solution (in DMSO at 10 μM and 100 μM) as required. Five replicate cultures were grown at each drug dose. Cells were cultured for 48 hours, and then harvested by cell scraper using 1 ml Trizol reagent (Cat. No. 15596-026, Invitrogen, UK) per flask, and stored overnight at -70°C. RNA was then precipitated and resuspended using an isopropanol precipation method [21]. cDNA was generated using random hexamer primers as described previously [22].

#### Sequencing

cDNA generated as above was used as template DNA for 35 cycles of PCR using the following conditions: 95°C (30s), 57°C (30s), 72°C (60s). To facilitate sequencing of large fragments, 2 overlapping fragments were amplified, covering the entire coding sequence, using the following primers:

Forward: 5'-GGCACAACCAGCACCATCT-3'

Reverse: 5'-CTCCACAGCAAATGATAGCAGC-3'

(Amplicon length 1185 bp, position 191-1375)

Forward: 5'-GCCAGTAACAATCCACCTTTT-3'

Reverse: 5'-AAGGTGTGGCTGATCTGAAGG-3'

(Amplicon length 735 bp, position 913-1647)

The PCR products were then subjected to electrophoresis on a 2% agarose gel. Bands corresponding to the above amplicons were then extracted from the gel using the Qiaquick kit according to the manufacturer's instructions (Cat. No. 28704, Qiagen, UK). This product was then used as template for a further 25 cycles of PCR, from which the relevant bands were again extracted and sequenced using the above primers on an ABI Prism 310 genetic analyzer (Applied Biosystems, UK). Sequence analysis and comparison was performed using DNAstar software (DNAstar Inc, USA).

#### Preparation of cDNA for microarray hybridisation

Following precipitation from Trizol, RNA was re-extracted using RNAeasy mini-columns (Cat. No. 74106, Qiagen, UK) and assayed for purity and concentration using spectrophotometry and an Agilent 2100 Bioanalyzer (Agilent, USA). Glass microarrays sourced from within the University of Cambridge Department of Pathology were printed on 2 slides and comprised over 10000 clones from various sources as previously described [22,23]. Confirmation of the array's performance had been conducted by in-house analysis of reproducibility [24], in addition to its use in prior validated experiments [23]. Further quality control of the arrays in this experiment was ensured by reproducibility analysis of the array data generated from the pooled DNA samples (see additional file 1). cDNA synthesis and labelling for hybridization was carried out as previously described with minor modifications [25]. 1 μg total RNA was used to synthesise double-strand cDNA (ds-cDNA) using the SMART PCR cDNA synthesis kit (Clontech, UK), according to the manufacturer's instructions, over 15 cycles of PCR.

#### Spotted oligonucleotide microarray hybridisation and scanning

The ds-cDNA was labelled by Cy3-deoxyuridine triphosphate or Cy5- deoxyuridine triphosphate (Amersham-Pharmacia, UK) using the Bioprime DNA labelling kit (Cat. No. 18094-011, Invitrogen, UK) with random hexamers. To counter the effect of dye bias, a reference cDNA consisting of amplified cDNA pooled from the five control-treated samples was labelled with Cy3. Each of the 15 sample cDNAs were labelled with Cy5. Paired samples (Pooled-Cy3 + Sample-Cy5) were purified using Autoseq G50 columns (Amersham, UK), mixed with 5 μg/ml Human Cot-1 DNA (Cat. No. 15279-011, Invitrogen, UK), 1 μg/ml Poly-dA (Amersham-Pharmacia, UK). Labelled targets were resuspended in 50 μl of hybridisation buffer (40% formamide, 5× SSC, 5× Denhardt's solution, 1 mM sodium pyrophosphate, 50 mM Tris pH 7.4, 0.1% SDS), denatured at 95°C for 5 min, incubated at 50°C for 5 min and then centrifuged at 8000 rpm for 5 min before being applied to the cover-slipped array. Hybridisations were performed at 50°C in a humidified environment for 16 h. Following hybridisation, slides were washed twice in 2× SSC for 10 min, twice in 0.1× SSC/0.1% SDS for 5 min and finally twice in 0.1× SSC for 5 min; all washes were performed at room temperature. After washing, slides were dried by centrifugation at 1000 rpm for 2 min, and then scanned using a Genepix 4100 microarray scanner (Axon Instruments, Foster City, CA). The scanned images were processed by using GenePix Pro 4.1 software (Axon).

#### Analysis of gene array data

Raw Cy3- and Cy5- channel data extracted using Imagene were normalized against a reference array using a LOESS-based algorithm with a span of 0.4 as previously described [13]. Empty spots and known housekeepers were removed from the data. Independent Component Analysis [26] was performed on the remaining data set. Components extracted were then compared against the array grouping using ANOVA in the R-statistical environment, to identify components related to drug dosage, where p < 0.01. Significant genes relating to high-dose fenofibrate were those with the top 1% of component loadings in the relevant component. Analysis of gene ontology (GO) classification was performed as previously described [13]

#### Quantitative Real-Time PCR (Taqman)

RNA samples analysed by RT-PCR were the same as those used for microarray analysis. Total RNA (2 μg) was incubated with random hexamer primers at 70°C and reverse-transcribed at 42°C using Super Reverse Transcriptase (HT Biotechnology Ltd, Cambridge, UK). 0.6 μl of template DNA was amplified using PCR MasterMix (Cat. No. AB-1142, ABgene, UK). All RT-PCR experiments were performed in an ABI Prism 7700 Sequence Detector (Applied Biosystems) in triplicate. Results for gene abundance in each sample were normalised to abundance of 18S RNA when appropriate. Normalised log-transformed transcript levels were compared across samples using one-way ANOVA and Student's t-tests.

#### (i) PPARα

Due to the low abundance of the PPARα transcript, 20 cycles of PCR (see below for conditions) were performed prior to the quantitative PCR cycles. The following primers were used to amplify a 553 bp region of PPARα which was subsequently amplified with Taqman reagents.

Forward: 5'-AGGGCCCTGTCTGCTCTGTG-3'

Reverse: 5'-CGGGTGGACTCCGTAATGATAG-3'

PCR product from this round of PCR was used as the template for subsequent quantitative PCR. Primer/probe sequences used for RT-PCR were:

Forward primer: 5'-GACGTGCTTCCTGCTTCATAGA-3'

Reverse primer: 5'-CACCATCGCGACCAGATG-3'

Probe: 5'-6FAM-TGGAGCTCGGCGCACAACCA-TAMRA-3'

#### (ii) Verfication of gene arrays

RT-PCR for three genes selected from microarray analysis was performed using the assays-on-demand primers below according to the manufacturers instructions (Applied Biosystems, USA).

(a) Cyclin D1 (CCND1) (Cat. No. Hs00277039_m1)

(b) Methionine adenosyltransferase II alpha (MAT2A) (Cat. No. Hs00428515_g1)

(c) Phosphoenolpyruvate carboxykinase 2 (PCK2) (Cat. No. Hs00356436_m1)

### Animal studies

All animal care and experimental protocols were approved by the animal ethics committee of the University of Cambridge and the Home Office of the United Kingdom government. 6-week old female cd-1 nude mice (Charles River Inc) were ovariectomised and implanted in the flank with one-half of a 1.5 mg 60-day release estradiol pellet (Cat. No. SE-121, Innovative Research of America, USA). After one week, mice that were to receive retinoic acid were implanted with a further pellet in the neck containing 5 mg ATRA (Cat. No. V-111, Innovative Research). At the same time, the diet was changed to a diet containing 0.1% or 0.25% fenofibrate (constituted by Lillico Biotechnology, Surrey, UK) or the same diet without fenofibrate. Approximately 1 million Ishikawa cells in 100 μl Matrigel (Cat. No. 35-6234, BD Biosciences, UK) were injected subdermally into the left flank, raising a bleb. Mice were checked daily and weighed twice-weekly to ensure good health. At the end of the experiment the mice were culled, the tumours were excised from surrounding tissue and weighed. Immunohistochemistry for PPARα was performed on selected harvested tumour samples as previously described [12]

#### Statistics

All statistical analyses were performed under the R statistical environment [27]. Where appropriate, Student's t-test, Wilcoxon Rank-Sum test or one-way ANOVA was used for comparisons as indicated. Methods particular to gene array analysis are described above.

## Results

### Growth inhibitory effect of fenofibrate is precipitated by retinoic acid

**Figure 1:**
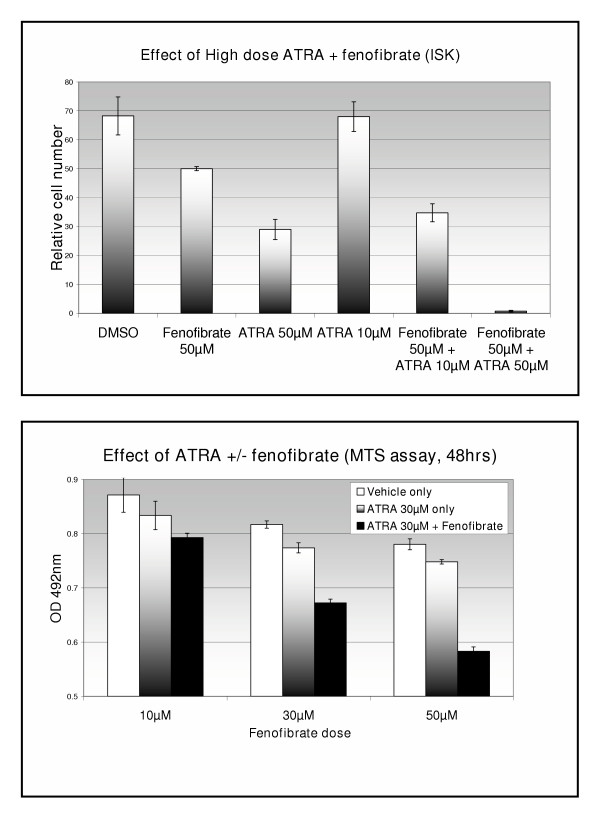
Effect of fenofibrate and retinoic acid (48 hours exposure) on endometrial cancer cell growth, using cell counting (*Top*) and MTS assay (*Bottom*).

Treatment of Ishikawa cells with 50 μM fenofibrate over 48 hours significantly reduced cell growth (27%, p < 0.05). Although treatment with 10 μM all-trans retinoic acid (ATRA) had no significant effect on cell growth, combined treatment with 50 μM fenofibrate caused a greater reduction in cell number than produced by fenofibrate alone (49%, p < 0.05). Treatment with high dose ATRA combined with fenofibrate caused >90% reduction in cell number (p < 0.05) (figure 1a). In addition, a dose-response was demonstrated when 10, 30 or 50 μM fenofibrate was combined with 30 μM ATRA (figure 1b). Similar effects were seen with retinoic acid, where no inhibition of cell growth was detected below 50 μM in the absence of fenofibrate (data not shown). BrdU assays again demonstrated inhibition of cell proliferation with fenofibrate but failed to show a similar effect with high dose rosiglitazone, a PPAR_γ _agonist. Other PPAR agonists and antagonists were tried but failed to show a significant effect in the doses used (data not shown)

### Fenofibrate induces cell cycle arrest and apoptosis in Ishikawa cells

**Figure 2:**
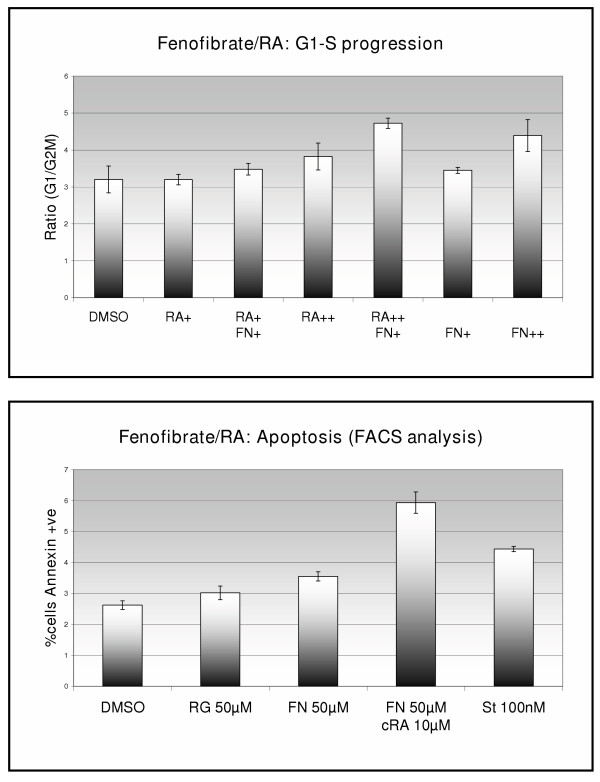
*Top *– DNA content analysis. The bars represent the ratio of cells in G1:G2M phase of the cell cycle after 12 hours of treatment with drug (RA+ = ATRA 10 μM. RA++ = ATRA 30 μM, FN+ = Fenofibrate 30 μM, FN++ = Fenofibrate 100 μM). *Bottom *– Apoptosis by FACS analysis using Annexin-FITC staining. Cells were treated for 3 hours. FN = fenofibrate, RG = rosiglitazone, St = staurosporine, a potent apoptogen.

The proportion of cells in G_1 _and G_2_M phases of the cell cycle were measured by FACS analysis after 12-hour exposure to fenofibrate. Application of 50 μM or 100 μM fenofibrate increased the ratio of cells in G_1 _phase to G_2_M phase of the cell cycle (data not shown, p < 0.05, Student's t-test). Treatment of ISK cells with 100 μM fenofibrate combined with 30 μM ATRA, however, had a greater effect on cell cycle progression than either drug alone (figure 2a). The effect of fenofibrate and retinoic acid on apotosis was also measured by FACS analysis, and similarly demonstrated an increase in apotosis induction when the drugs were combined (figure 2b).

**Figure 3:**
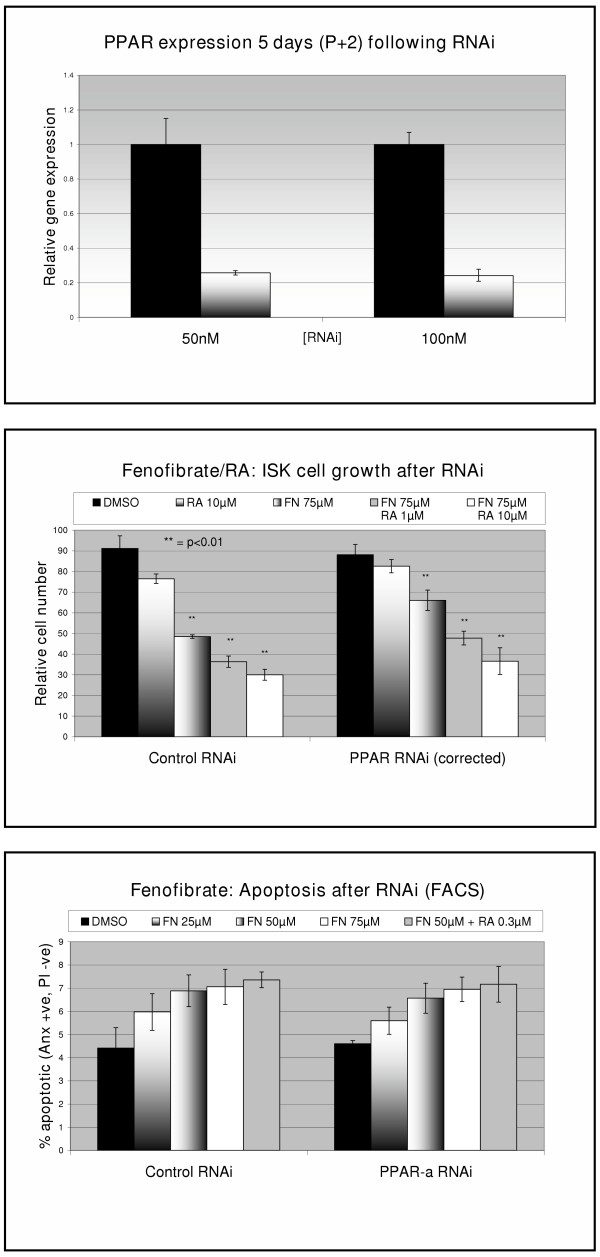
*Top *– mRNA expression as quantified using Taqman RT-PCR following RNAi for PPARα using RNAi concentration shown. *Middle *– Cell growth following RNAi and treatment with fenofibrate (FN) and ATRA (RA) for 48 hours. Cell numbers were corrected to adjust for the reduced cell growth following PPARα RNAi compared to control RNAi. *Bottom *– Apoptosis measured by FACS analysis using Annexin-FITC staining following RNAi and treatment with fenofibrate and retinoic acid for 3 hours.

Although our previous results had suggested a direct effect of fenofibrate on the PPARα receptor, experiments conducted using RNAi for PPARα failed to show a dramatic reduction in fenofibrate effect. This was despite achieving consistent downregulation of >75% in PPARα expression as measured by RT-PCR (Figure 3a). The effect of ATRA combined with fenofibrate on cell growth was similar in the presence or absence of PPARα RNAi (figure 3b). However, the total viable cell number in the PPARα^- ^transfected control-treated cells was approximately 40% lower than the control RNAi transfected cells. After correcting for this difference, the effect of 75 μM fenofibrate (48 hours treatment) on cell growth inhibition was less in the RNAi transfected cells (p = 0.05, t-test) (figure 3b).

### Genes affected by fenofibrate in Ishikawa cells

Using Independent Component analysis (ICA), a component relating to treatment with high-dose fenofibrate (100 μM) was identified in both gene array experiments (slide 1 & slide 2) (p < 0.01 after correction for multiple testing. Other significant components identified from ICA related to batch effects (hybridisation date, RNA preparation date, hybridisation batch) (p < 0.01, ANOVA). Batch effects were so strong that the "dose" component was only the 9^th ^largest of 30 components. No component could be identified relating to "low dose" (10 μM) fenofibrate treatment.

**Figure 4:**
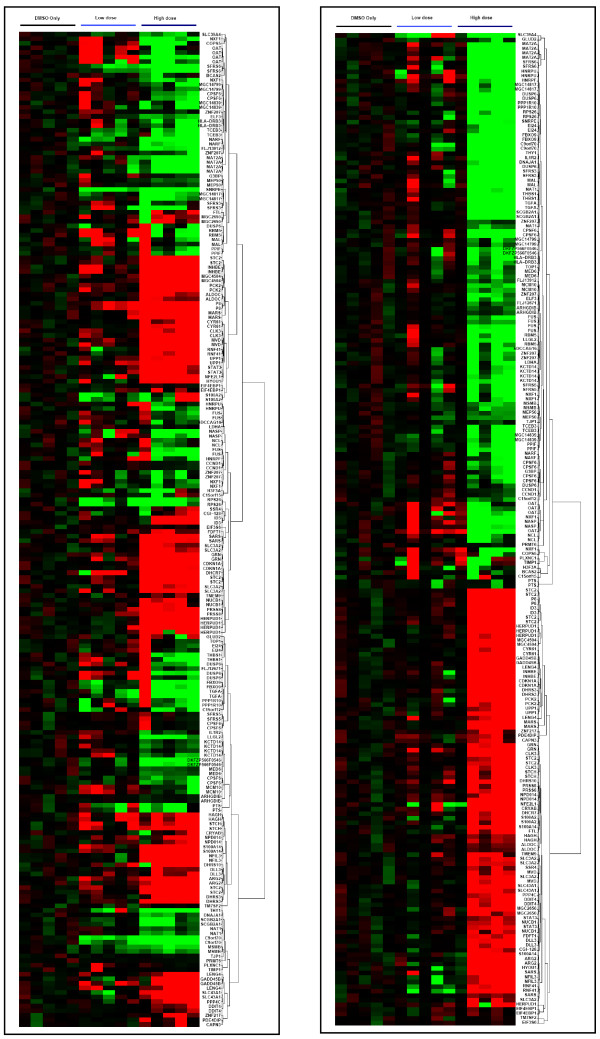
Genes identified as differentially expressed after treatment with high-dose (100 μM) fenofibrate. The plots show upregulation (red) and downregulation (green) of expression compared to DMSO control-treated cells. Cells were treated with 10 μM ("low dose") or 100 μM ("high dose") fenofibrate for 48 hours. *Left *– Normalised uncorrected data. *Right *– Data corrected using ICA-based filtering and removal of artefactual components. The data is improved but reveals an aberrant sample in the high-dose group (the first).

Significant genes from the identified component were selected as those with absolute component loading values greater than 3 standard deviations, treating positive and negative values separately. This was an arbitrary value providing a reasonable number of genes all of which had component loadings outside the inflexion of the plotted curve (see additional file 1). At this threshold, 425 gene spots representing 213 annotated unique genes from Slide 1 were identified as differentially expressed following treatment with high dose fenofibrate. A similar number of differentially expressed genes were additionally identified from the second microarray experiment (using Slide 2 which contained a greater number of non-anottated clones). A subset of genes from the Slide 1 geneset is displayed as an expression plot in figure 4a.

**Figure 5:**
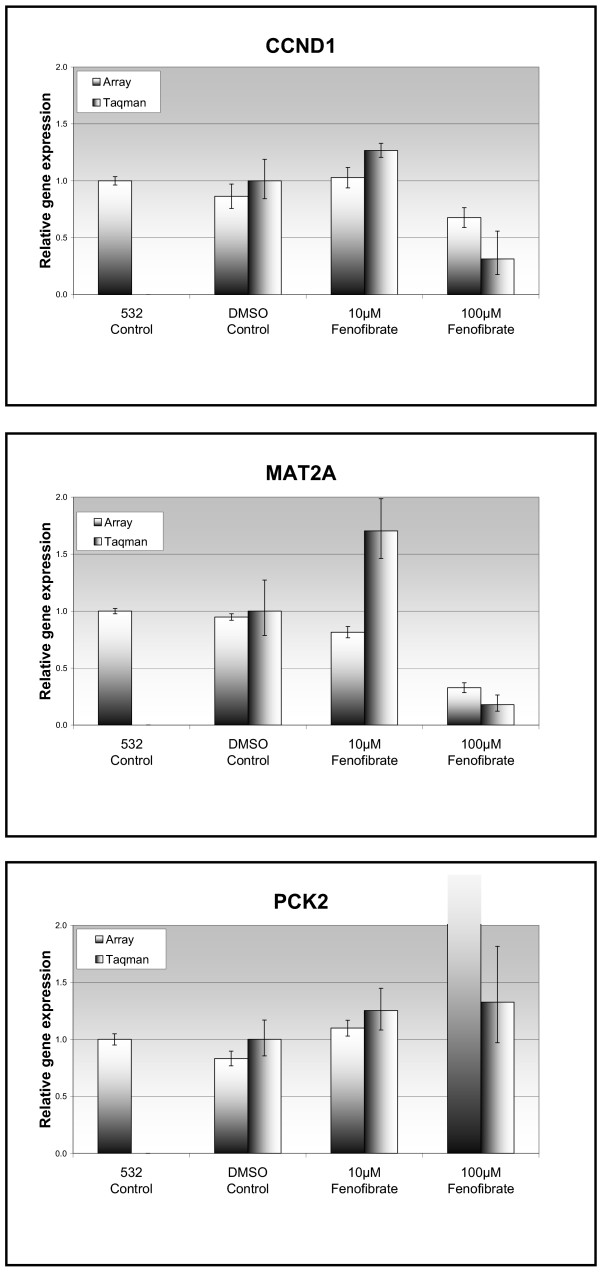
Comparison between array data and gene expression (as measured using Taqman RT-PCR). The "532 control" column represents the green-channel array data from the pooled cDNA (from DMSO-treated control samples) and is used as an internal control. Three genes were chosen for verification of the array: *top *– Cyclin D1, *middle *– Methionine Adenosyltransferase 2-alpha, *bottom *– Phosphoenolpyruvate carboxykinase 2.

Three genes were chosen for RT-PCR validation of the gene array (figure 5): Cyclin D1 (CCND1), Phosphoenol pyruvate carboxykinase 2 (PCK2) and Methionine Adenosyltransferase 2A. These genes were chosen due to a combination of their loading value in the component of interest (suggesting a significant effect) as well as their biological interest. Both CCND1 and MAT2A were downregulated following treatment with fenofibrate 100 μM. RT-PCR analysis confirmed downregulation in gene expression (CCND1 3.2-fold, MAT2A 5.6-fold, p < 0.05). RT-PCR analysis confirmed a small increase in PCK2 expression (1.3-fold, p = NS) in the treated cells but this was less than that seen with the array data (figure 5c).

Gene Ontology analysis revealed a number of functional groups which were significantly over-represented in the transcripts identified as altered by fenofibrate. A functional group was considered to be over-represented in the gene list when the number of gene spots belonging to that group was higher than expected from the distribution of genes on the array (chi-squared test). The following functional groups of interest were identified as being over-represented by the genes altered with fenofibrate treatment (see table 1): nucleotide binding (GO:0000166), MAP kinase-related (GO:0017017, GO:0000188, GO:0000185), cell growth (GO:0016049, GO:0008283), cyclin-dependent protein kinase inhibitor (GO:0004861), insulin-like growth factor binding (GO:0005520), platelet-derived growth factor receptor ligand (GO:0005161), metalloprotease inhibitor (GO:0008191) and cell cycle arrest (GO:0007050). The full table of GO classifications, by significance level, is provided in additional file 2.

**Table 1:** Selected genes derived from ICA & GO analysis of gene array experiments. Genes are combined into ontologies. Average Fold Changes are calculated from normalised values over the number of spots shown for each gene. As can be seen, not all genes are differentially expressed according to t-test p-values. Genes are instead selected based on their ICA loading (see discussion)

**GO Description**	**Gene symbol**	**Description**	**No of spots**	**Ave FC**	**Ave ttest p**
activation of MAPKKK (GO:0000185)	GADD45B	growth arrest and DNA-damage-inducible, beta	2	2.6	0.090
cell cycle arrest (GO:0007050)	EIF4G2	eukaryotic translation initiation factor 4 gamma, 2	2	1.2	0.249
	GADD45A	growth arrest and DNA-damage-inducible, alpha	2	5.1	0.003
	PPP1R15A	protein phosphatase 1, regulatory (inhibitor) subunit 15A	2	1.4	0.085
	PR48	protein phosphatase 2A 48 kDa regulatory subunit	1	-1.1	0.427
	SESN3	sestrin 3	2	-1.3	0.161
	TP53	tumor protein p53 (Li-Fraumeni syndrome)	4	1.7	0.036
cell death (GO:0008219)	EIF4G2	eukaryotic translation initiation factor 4 gamma, 2	2	1.2	0.249
	EMP3	epithelial membrane protein 3	2	1.7	0.111
cell growth (GO:0016049)	P8	p8 protein (candidate of metastasis 1)	2	3.1	0.005
	SLC3A2	solute carrier family 3 (activators of dibasic and neutral amino acid transport), member 2	4	2.0	0.012
cell proliferation (GO:0008283)	EGFR	epidermal growth factor receptor (erythroblastic leukemia viral (v-erb-b) oncogene homolog, avian)	2	2.2	0.009
	FTH1	ferritin, heavy polypeptide 1	1	1.2	0.301
	IGFBP4	insulin-like growth factor binding protein 4	2	1.8	0.002
	OSMR	oncostatin M receptor	2	1.7	0.088
	PDGFA	platelet-derived growth factor alpha polypeptide	2	1.5	0.032
	PDGFB	platelet-derived growth factor beta polypeptide (simian sarcoma viral (v-sis) oncogene homolog)	4	2.2	0.083
	SYK	spleen tyrosine kinase	2	-1.1	0.631
	TGFA	transforming growth factor, alpha	2	-1.0	0.907
	TGFBI	transforming growth factor, beta-induced, 68kDa	2	2.1	0.164
	TP53	tumor protein p53 (Li-Fraumeni syndrome)	4	1.7	0.036
	VEGF	vascular endothelial growth factor	2	6.6	0.175
G1/S transition of mitotic cell cycle (GO:0000082)	CCND1	cyclin D1 (PRAD1: parathyroid adenomatosis 1)	2	-1.3	0.168
insulin-like growth factor binding (GO:0005520)	CYR61	cysteine-rich, angiogenic inducer, 61	2	2.9	0.013
	KAZALD1	Kazal-type serine protease inhibitor domain 1	2	-1.1	0.770
	IGFBP4	insulin-like growth factor binding protein 4	2	1.8	0.002
	CTGF	connective tissue growth factor	2	2.9	0.006
	IGFBP5	insulin-like growth factor binding protein 5	4	2.7	0.041
metalloprotease inhibitor (GO:0008191)	TIMP1	tissue inhibitor of metalloproteinase 1 (erythroid potentiating activity, collagenase inhibitor)	3	1.4	0.250
methionine adenosyltransferase (GO:0004478)	MAT2A	methionine adenosyltransferase II, alpha	4	-2.9	0.001
methionine-tRNA ligase (GO:0004825)	MARS	methionine-tRNA synthetase	2	6.7	0.006

### Effect of fenofibrate on tumour growth in vivo

**Figure 6:**
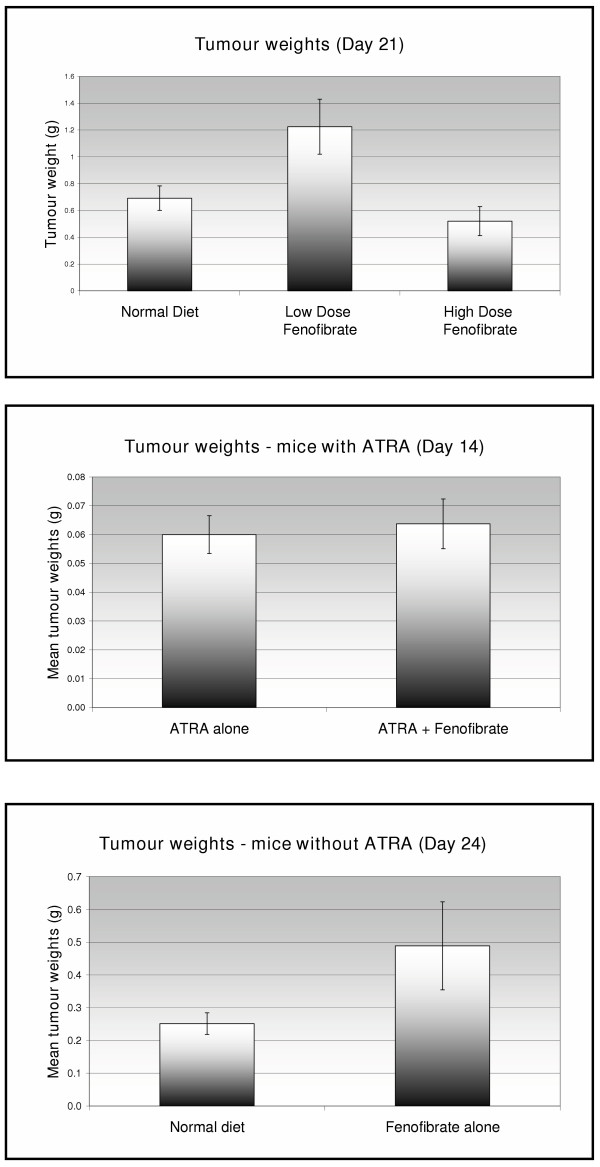
Tumour weights in nude mice following endometrial cancer cell xenografting and treatment with "high dose" (0.25%) or "low dose" (0.1%) fenofibrate. *Top *– n = 6 mice per group treated for 21 days. *Middle *– n = 8 mice per group treated for 14 days and *Bottom *– n = 8 mice per group treated for a further 10 days with fenofibrate alone. Tumour weights were lower in the high-dose fenofibrate-treated mice in the first experiment, but this was not repeated in the second experiment.

Two separate *in vivo *experiments were performed (figure 6). In the first, mice were allocated to each of three groups – (1) normal diet (N = 6), (2) low-dose fenofibrate (0.1%) (N = 4), (3) high-dose fenofibrate (0.25%) (N = 6). The median tumour weights at 21 days in the three groups were (1) 684 g (2) 1280 g (3) 439 g. There was a trend towards lower tumour weights in the high-dose-treated group compared to those receiving normal diet (p = 0.046, Mann-Whitney test, one-sided). Surprisingly, however, the group fed low-dose fenofibrate demonstrated the largest tumours at 21 days (p < 0.05, Mann-Whitney test).

In the second experiment, 8 mice were allocated to each of four groups, to receive (1) normal diet, (2) high-dose fenofibrate (0.25%), (3) ATRA, (4) ATRA + fenofibrate. All mice treated with ATRA were culled at day 14 due to weight loss (>10% starting weight) and the majority displayed a local reaction to the ATRA pellet. The remaining mice were culled at day 28. There were no significant differences between the tumour weights in the groups compared (fenofibrate vs normal diet; ATRA vs ATRA + fenofibrate) (Mann-Whitney test, p > 0.05)

## Discussion

To our knowledge, we are the only group to have published on the effects of fenofibrate in endometrial cancer in vitro [12]. Our previous data demonstrated a moderate inhibitory effect of fenofibrate on endometrial cancer cell growth but with limited understanding of the mechanism of this effect. This study significantly expands on this knowledge.

We have confirmed, using additional methods, that Ishikawa endometrial cancer cell growth is inhibited by fenofibrate. We have also confirmed, by using FACS analysis, that fenofibrate increases apoptosis in ISK cells *in vitro*. These effects appear not to be associated with excess toxicity. More importantly, we were able to show, using drugs acting on the same pathway, that the inhibitory effects on cell growth can be potentiated using agents acting on the same pathway. The use of either all-trans-retinoic acid (ATRA) or its metabolite 9-cis retinoic acid in this regard demonstrated similar results, as might be expected. Furthermore it might be anticipated that these agents potentiate the effect of fenofibrate via their action on the PPARα receptor [28]. Similarly, a synergistic effect of RXR and PPAR agonists has previously been demonstrated in breast cancer [29] and bladder cancer [30] cell lines. However, although retinoic acid derivatives alone have been demonstrated to show activity in breast cancer [31,32] and other tumours, particularly leukaemias [33,34], there is limited evidence that retinoids are effective in endometrial cancer [15,35]. Indeed, our data suggest that, even at comparatively high doses of retinoic acid, no inhibition of Ishikawa cell growth occurs.

The mechanism of action of fenofibrate is, as yet, unknown. A parallel might be drawn between the effect of fenofibrate (a PPARα agonist) in endometrial cancer and thiazolidenediones (TZDs, PPAR_γ _agonists), such as rosiglatazone and troglitazone, in breast cancer. TZD's have been demonstrated to cause inhibition of cancer cell growth *in vitro *and/or *in vivo *in a number of cancers, particularly breast [29,36], colon [37] and salivary gland [38]. Although yet to be fully determined, it would appear that at least one mechanism for this action involves inhibition of translation and transition through G_1_-S phase of the cell cycle39. These effects are associated with decreased expression of D1/E cyclins in the absence of a change in p21, or cyclin-dependent kinases (CDK's) and are independent of PPAR_γ _activation [39].

Anti-tumour effects of PPARα-specific agonists appear to be less commonly reported. One reason for this may be the perception that PPARα agonists are carcinogenic due to their tumorigenic effects in rodent liver [14]. Furthermore, agonism of PPARα has been shown to increase proliferation in MCF-7 breast cancer cells [40]. This effect, however, is reported inconsistently and PPARα agonists have also demonstrated efficacy *in vitro *against melanoma [41] and breast cancer cell lines [42]. These studies outline various mechanisms which explain the effect of fenofibrate on cancer cells. Firstly, PPARα agonist effects on the cell cycle and apoptosis are mediated by environmental agents and the MAP kinase pathway [43]. PPAR activity is also closely linked to the eicosanoid pathway of inflammation and PPARα induces cyclo-oxygenase-2 (PTGS2) expression, which is linked to the development of colon cancer [44]. Furthermore, there is significant cross-talk between PPAR receptors and many other nuclear hormone receptor subfamilies [45-47]. The most obvious of these is the link to the retinoid receptor family, but even within this subfamily, sex-steroid hormone influence is evident. Examples of this are the estrogen-dependence of retinoic acid metabolism [48] and the fact that PPARα-mediated anti-inflammatory action stimulated by TNFα is interrupted by the anti-progesterone RU486 in HUVEC's [49]. Probably the strongest nuclear hormone interrelationship with the PPAR receptors is to insulin and the IGF-receptor [47,50,51]. Although the primary clinical function of TZD's is to increase insulin sensitivity, PPARα agonists also reduce insulin resistance and insulin concentration *in vivo *[52]. Furthermore, the strong association between PPARs and lipid and glucose homeostasis appears to be a primary factor in the development of obesity [47,53]. Taken together, these features support the hypothesis that the PPAR pathway might harbour anti-neoplastic targets in endometrial cancer, which is known to be promoted by obesity and hyperinsulinaemia [5]. Indeed, the inhibition of G_1_-S phase progression, demonstrated in this study with fenofibrate, is consistent with the anticipated effects of targeting of PPARα in endometrial cancer.

We attempted to demonstrate that the inhibitory effect of fenofibrate on Ishikawa cell growth was specific for PPARα. Support for this hypothesis was provided by the synergistic effects demonstrated from combined treatment with fenofibrate and retinoic acid, and from the lack of similar effect seen with therapeutic doses of rosiglitazone. We have previously demonstrated a functional PPARα receptor using a luciferase reporter assay [12]. In this study we also demonstrated absence of mutation within the coding region of the hPPARα gene in the cell lines tested, as it is known that up to 15% of endometrial cancers harbour variants of PPAR_γ _[54].

The gene array experiments were designed to better understand the mechanism of action of this drug, at the transcript level. The traditional approach to identification of differentially expressed genes involves the use of first-order statistics such as the t-test (eg Cyber-T [55]) or permutation-based methods (eg SAM [56]), often in combination with a "threshold of interest" applied to fold-change values in gene expression. Here we have applied Independent Component Analysis (ICA) [26,57] to identify a "gene signature" which correlates with our biological feature of interest, ie response to high-dose fenofibrate. Using this method we are not only able to filter out stochastic noise from the microarray data, but also to exclude components of the data associated with experimental artefact such as batch effects. Using ICA we could not discern a gene signature associated with the response to low-dose (10 μM) fenofibrate, suggesting that the effects on the transcriptome at this dose were small, consistent with the lack of biological effect seen. Of more interest, however, was the response to high-dose (100 μM) fenofibrate, which was seen in a significant proportion of the transcriptome (see figure 4). One advantage of using a factor analysis method is the avoidance of supervision bias when identifying differentially expressed genes. Here we combined ICA with Gene Ontology [58] analysis to identify groups of genes with shared function which were differentially expressed with high-dose fenofibrate.

Amongst the identified gene ontology groups, ontologies related to cell proliferation were over-represented in the list of differentially expressed genes. These included cell proliferation, cell growth, cell cycle arrest, and ontologies related to MAPkinase signalling. Within these functional groups, genes that were up- or down-regulated were generally consistent with the experimental findings of inhibition of G_1_-S phase progression. Downregulation of Cyclin D1 (CCND1) was accompanied by upregulation of p8, p21, GADD45A, GADD45B and TP53. Cyclin D1 is known to be upregulated in endometrial cancer and is a common feature of endometrial carcinogenesis [59,60]. Cyclin D1 plays a major role in cell cycle progression and is therefore central to the proliferative ability of many cancers [61]. In view of its importance in cell cycle progression, we chose Cyclin D1 as one of the genes with which to verify the array findings and confirmed its downregulation (see figure 4b). Conversely, and as might be expected, p21 (cyclin-dependent kinase inhibitor 1a, CDKN1A) was upregulated following administration of high-dose fenofibrate. P8 (COM-1) was also upregulated. P8 encodes for a protein which inhibits cancer cell growth in estrogen-dependent breast cancer cell lines [62]. TP53 is another protein central to the regulation of cancer cell growth [63] and its gene expression was also upregulated.

Although cell cycle-related genes are important anti-neoplastic targets, other pathways are particularly relevant in endometrial carcinogenesis. There is overwhelming evidence that obesity plays a pathogenetic role in endometrial cancer and is most likely mediated by IGF [5,7]. In particular, the IGF binding proteins appear to play a pivotal role in estrogen-dependent endometrial cancer cell growth [64]. Transcript levels of both IGFBP4 and IGFBP5 as well as Connective Tissue Growth Factor (CTGF, IGFBP8) were increased in response to fenofibrate, suggesting that this lipid-lowering agent may exert some direct action on pathways known to be relevant to endometrial carcinogenesis.

The gene array experiments also revealed a further pathway which may be involved in the inhibition of Ishikawa endometrial cancer cell growth. Two of the genes that were most differentially regulated by fenofibrate were methionine adenosyltransferase 2-alpha (MAT2A) (FC = -2.7, p < 0.01) and methionine-tRNA synthetase (FC = 6.7, p < 0.01). These two genes form part of the methionine metabolism pathway, central to normal cellular function and DNA synthesis. In particular, MAT2A catalyses the conversion of L-Methionine to S-Adenosyl methionine, the principal methyl donor. Induction of MAT2A is mediated by TNFα and reduced expression is associated with inhibition of cell growth [65,66]. Induction of MAT2A has also been correlated with disease progression in colon cancer [67]. MAT2A may also prove to be an important therapeutic target in view of the increasingly realised epigenetic role of methylation in endometrial carcinogenesis [68,69]. By contrast, whether MARS plays a role in carcinogenesis is not known, and the induction of this gene may simply be a reflection of the degree of translational change taking place under the influence of fenofibrate as suggested earlier [39].

In addition to gene array analysis, we applied RNA-interference [70] in order to establish whether the inhibitory effects of fenofibrate seen were due to activation of the PPARα receptor. Although our results demonstrated some diminution of effect at lower doses of fenofibrate, the main effects were unchanged despite confirmation of at least 75% knock-down of PPARα expression. A further unexpected effect encountered during the RNAi experiments was the significant and consistent reduction in cell viability seen following knock-down of PPAR expression. This might be explained by the central role PPARα plays in cellular metabolism and may also account for a blunting of any direct effect on cell growth induced by PPARα knock-down.

Furthermore, it was expected that some of the differentially expressed genes identified in the array experiments would comprise those known to be regulated by fenofibrate. Comparison of our gene list to previously published data [71,72] failed to demonstrate a similar pattern of differentially expressed genes, although there were occasional similarities (eg ↑Stearoyl-CoA desaturase). One explanation for this may be that the small changes in metabolic gene expression remained undetectable in an environment of apoptosis and cell death. A second explanation, however, could be that the majority of published gene expression data relating to fenofibrate in rodents is less applicable to human cells.

This latter possibility may also explain the absence of a consistent demonstrable effect on tumour growth *in vivo*. Results from our initial experiment in mice were encouraging, but failed to show reduction in tumour growth, or a difference when retinoic acid was added, in the second experiment. Furthermore, immunohistochemistry failed to identify significant levels of PPARα protein expression in the harvested tumour samples, raising the possibility that the receptor level is not maintained within the animal model environment (data not shown). There are known species differences in the effects of fenofibrate: humans have a lower level of liver PPARα expression than rodents [73], the promoter for human Acyl CoA Oxidase is different from that in rodents [74] and effects on the cell cycle in mice liver are different from that in humans [75]. Taken together, these findings may explain the unexpected results from our *in vivo *experiments. A further unexpected finding from the animal experiments was the increased tumour size seen in the low dose fenofibrate arm compared to normal diet (p = 0.04). It was postulated that the serum level of fenofibrate in the low dose arm may have been well below the anticipated dose, and that a low fenofibrate dose may have had a stimulatory effect on ISK cell growth. A further experiment was therefore conducted to investigate the effect of prolonged (>96 hours) low-dose (0.1 μM – 3 μM) exposure to fenofibrate, but failed to show any effect on cell growth (data not shown). The increased tumour size in this group was therefore considered to be a statistical, rather than a true biological effect. A further possibility explaining the absence of expected effect may have been inadequate dosing, although the doses chosen were based on previously published data to achieve desired serum fenofibrate levels [76]. One further point to note was that the mechanism for delivery of ATRA ultimately proved to be a problem resulting in toxicity, either due to the dose (although this was well within established dosage according to the manufacturer) or to local reaction to the pellet. Our preference would have been to use Liposomal ATRA (ATRA-iv, Antigenics Inc, New York) but it proved impossible to obtain this from the manufacturer. It is possible that the toxicity encountered from the pellets prevented a demonstrable response to fenofibrate in the final experiment.

PPAR_γ _agonists have been demonstrated to show tumour inhibition in vitro and in vivo, in a number of tumour types such as breast and colon. PPARα agonists, however, have so far failed to show similar results, despite inhibitory effects on tumour growth in breast cancer [42] and melanoma [41] in vitro. It is highly plausible that the species differences in PPARα activity [72,77] is at least in part responsible for the difficulties in demonstrating efficacy of PPARα agonists in a rodent model of human tumours. The promising effects of these agents in vitro, however, suggest that the lack of a consistent effect on endometrial cancer in mice warrants further investigation, most likely requiring the use of an alternative animal model.

The full mechanism of action of fenofibrate remains to be clarified but it appears to exert effects on cell cycle regulators in addition to pathways pertinent to endometrial carcinogenesis. The data presented suggest that PPARα remains a potential therapeutic target in endometrial cancer, whilst the addition of retinoic acid to fenofibrate creates a potent therapeutic combination *in vitro*. The development of an appropriate animal model, however, remains essential to demonstrating the applicability of this promising combination in the treatment of endometrial cancer.

## Competing interests

The author(s) declare that they have no competing interests.

## Supplementary Material

Additional File 1 Contains supplemental figures referred to in the main text. The main text is self-contained and does not required these figures which are provided for further reference for the interested reader Click here for file

Additional File 2 Contains a supplemental table referred to in the text and is an expansion of table 1 (from the main paper). Click here for file
