# Supplementary material for: In vitro and in vivo effects of the PPAR-alpha agonists fenofibrate and retinoic acid in endometrial cancer
Source: Mol Cancer. 2006 Mar 28;5:13. doi: 10.1186/1476-4598-5-13 (PMC1475879; doi:10.1186/1476-4598-5-13)
Supplement: Additional File 1 — Contains supplemental figures referred to in the main text. The main text is self-contained and does not required these figures which are provided for further reference for the interested reader [file 1476-4598-5-13-S1.pdf]

Supplementary Information

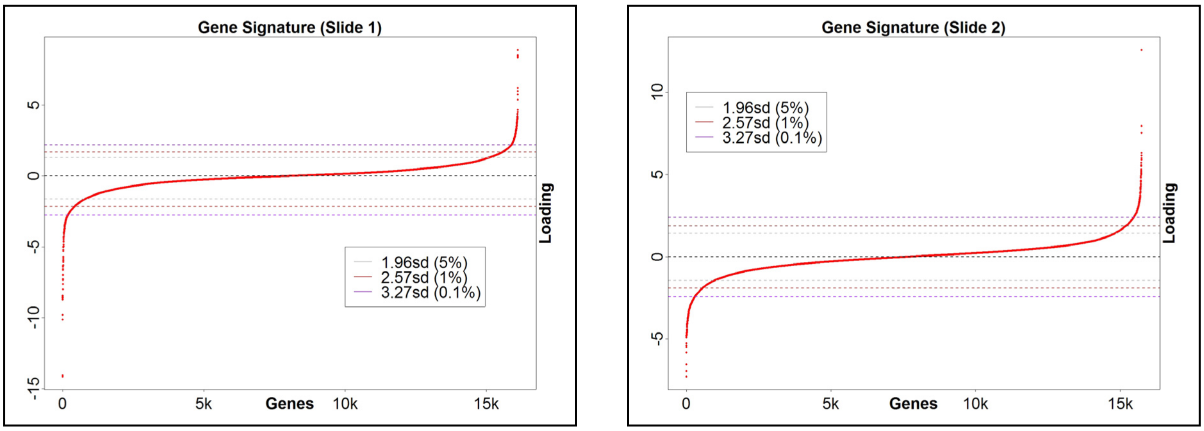

Figure 1: Identification of significant genes based on component loadings for the ICA component of interest. Genes with loadings lying outside the 0.1% (3.27sd) line are considered to be significantly differentially expressed. This threshold corresponds the inflexion of the loading curve plotted as above

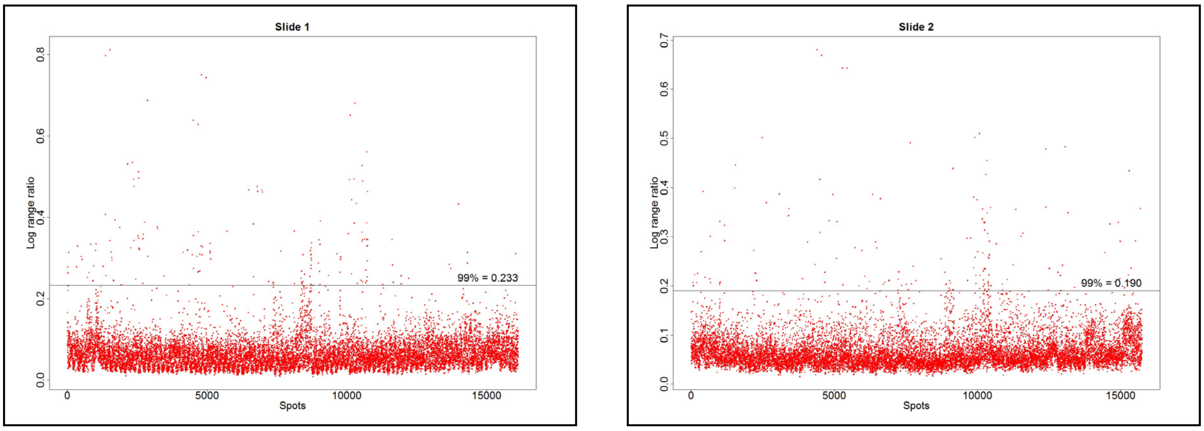

Figure 2: Quality Control validation of gene arrays. The standard deviation of the $\log_{10}$ signals from the 15 control gene arrays (hybridised from the same pooled DNA, Cy3 channel) are plotted for each slide. For each slide, the 99th centile of the standard deviations are also plotted (horizontal line). 99% of the spots demonstrate a calculated range ratio of ≤1.7 (Slide 1) or ≤1.6 (Slide 2). The range ratio signifies the ratio of the "estimated highest signal" to "estimated lowest signal" (ISO5725 reproducibility limits) for each spot, derived from the 95% Confidence Interval of the logged signal ($10^{4s/\sqrt{n}}$ where n=15 samples, s=standard deviation of log signal).
